# Supplementary material for: Evolving antibody evasion and receptor affinity of the Omicron BA.2.75 sublineage of SARS-CoV-2
Source: iScience. 2023 Oct 18;26(11):108254. doi: 10.1016/j.isci.2023.108254 (PMC10654603; doi:10.1016/j.isci.2023.108254)
Supplement: Document S1. Figures S1, S2, Tables S1, and S2 [file mmc1.pdf]

iScience, Volume *26*

Supplemental information

Evolving antibody evasion and receptor affinity of the Omicron BA.2.75 sublineage of SARS-CoV-2

Qian Wang, Zhiteng Li, Yicheng Guo, Ian A. Mellis, Sho Iketani, Michael Liu, Jian Yu, Riccardo Valdez, Adam S. Lauring, Zizhang Sheng, Aubree Gordon, Lihong Liu, and David D. Ho

A

Mutations on top of D614G	Alpha	Beta	Gamma	Delta	BA.1	BA.1.1	BA.2	BA.2.12.1	BA.4/5	BF.7	BA.4.6	BQ.1	BQ.1.1	XBB	XBB.1	XBB.1.5	BA.2.75	BL.1	BM.4.1	BM.4.1.1	BN.1	CH.1	CH.1.1	DS.1	BA.2.75.1	BA.2.75.2	BA.2.75.4	BA.2.75.5	BA.2.75.6	BA.2.75.7
R346T						T				T	T		T	T	T	T		T		T	T	T	T	T		T			T	
K444T												T	T									T	T							
L452Q/R				R				Q	R	R	R	R	R										R				R			
F486V/P/S									V	V	V	V	V	S	S	P			S	S		S	S	S		S				S
F490S														S	S	S					S			S						
D574V																		V							V					
K356T																					T			T				T		
R403K																								K						

B

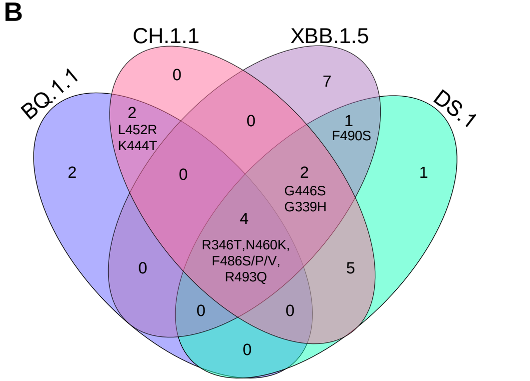

Figure S1. Key spike mutation in BA.2.75 subvariants. (A) Summary of spike mutations in SARS-CoV-2 VOCs. The convergent mutations are shown in blue and unique mutations among BA.2.75 subvariants are shown in orange. **(**B) The Venn diagram shows the number of shared spike mutations among BQ.1.1, XBB.1.5, CH.1.1, and DS.1 compared to BA.2. The RBD mutations shared by at least two subvariants are displayed. Related to **Figure 1**.

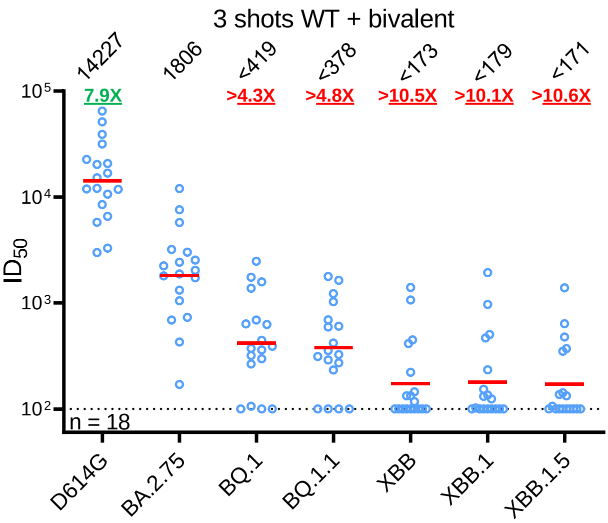

Figure S2. Neutralization of pseudotyped SARS-CoV-2 subvariants by polyclonal sera from "3 shots WT + bivalent" cohort. "3 shots WT + bivalent" refers to individuals vaccinated with three doses of the wildtype mRNA vaccine and subsequently one dose of a WA1/BA.5 bivalent mRNA vaccine. The results are shown as dots with geometric mean (red line). Values above the dots denote the raw geometric mean ID_{50} values and the sample size (n) is shown on the lower left. The limit of detection is 100 (dotted line). Comparisons were made against BA.2.75 and the fold changes in ID_{50} values are shown, with resistance to neutralization highlighted in red and sensitization in green. Statistically significant fold changes (p <0.05, determined by using two-tailed Wilcoxon matched-pairs signed-rank tests) are highlighted in bold. Related to **Figure 4**.

Table S1. Neutralization IC_{50} values for indicated pseudotyped D614G, BA.4/5, BA.2.75, and BA.2.75 subvariants by mAbs. Related to **Figure 3**.

IC_{50} (μg/ml)		NTD	NTD-SD2	SD1	RBD mAbs																											Combinations	
					Class 1					Class 2					Class 3																Class 4		
Pseudo-viruses	Spike mutations in the background of BA.2.75	C1520	C1717	S3H3	Brii-196	Omi-3	Omi-18	BD-515	COVOX-222	XGv051	XGv347	ZCB11	S2E12	COV2-2196	LY-CoV1404	2-7	XGv289	XGv264	S309	P2G3	SP1-77	BD55-5840	BD55-3152	XGv282	BD-804	A19-46.1	35B5	JMB2002	Brii-198	COV2-2130	10-40	COV2-2196 + COV2-2130	Brii-196 + Brii-198
D614G	NA	0.004	0.293	0.033	0.022	0.009	0.023	0.019	0.017	0.001	0.002	0.003	0.001	0.002	0.002	0.005	0.002	0.001	0.027	0.001	0.004	0.002	0.003	0.000	0.016	0.013	0.018	0.006	0.250	0.008	0.085	0.004	0.039
BA.4/5	NA	0.002	0.218	0.030	0.904	0.049	0.014	0.010	0.113	0.108	2.429	3.347	>10	0.842	0.001	0.066	0.041	0.003	0.577	0.002	0.007	0.019	0.071	0.001	0.022	7.765	>10	4.148	>10	0.028	0.806	0.070	0.676
BA.2.75	NA	0.004	0.303	0.025	1.418	0.064	0.028	0.127	2.000	0.002	0.002	0.003	0.017	0.032	0.005	1.959	0.074	0.006	0.436	0.003	0.009	0.598	0.152	0.009	0.048	0.097	0.611	0.091	0.989	0.044	3.346	0.046	0.992
BL.1	R346T/D574V	0.005	0.358	0.021	7.219	0.211	0.129	0.302	>10	0.003	0.001	0.006	0.014	0.052	0.004	1.978	0.176	0.009	0.325	0.438	>10	2.464	>10	0.025	7.836	0.804	7.501	>10	>10	>10	2.912	0.163	2.581
BM.4.1/BA.2.75.7	F486S	0.003	0.334	0.015	7.726	1.797	0.585	0.950	>10	0.909	>10	>10	>10	>10	0.005	1.967	0.132	0.005	0.254	0.003	0.005	0.728	0.140	0.008	0.045	0.098	0.601	0.090	1.616	0.042	2.665	0.080	2.233
BM.4.1.1	R346T/F486S	0.002	0.390	0.011	4.461	1.450	0.417	0.520	>10	0.472	>10	>10	>10	2.584	0.002	1.251	0.077	0.009	0.280	0.204	>10	>10	>10	0.004	0.939	0.355	>10	>10	6.341	>10	1.326	>10	2.150
BN.1	R346T/K356T/F490S	0.002	0.341	0.011	3.564	0.168	0.049	0.131	>10	0.002	0.001	0.004	0.018	0.073	0.002	2.381	0.059	0.004	5.208	0.164	>10	>10	>10	0.019	>10	>10	>10	>10	>10	>10	3.717	0.215	2.347
CH.1	R346T/F486S/K444T	0.002	0.314	0.010	>10	0.555	0.332	0.488	6.302	0.510	>10	>10	>10	>10	>10	>10	0.338	>10	0.291	>10	>10	>10	>10	0.132	>10	0.570	>10	>10	>10	>10	6.003	>10	>10
CH.1.1	R346T/F486S/L452R/K444T	0.002	1.089	0.011	>10	1.611	0.496	0.635	>10	>10	>10	>10	>10	>10	>10	>10	1.269	>10	0.437	>10	>10	>10	>10	2.529	>10	>10	>10	>10	>10	>10	>10	>10	>10
DS.1	R346T/K356T/R403K/F486S/K444T	0.002	0.403	0.015	>10	1.289	0.642	2.312	>10	>10	>10	>10	>10	>10	0.002	3.870	0.045	0.003	>10	0.409	>10	>10	>10	0.012	>10	>10	>10	>10	>10	>10	6.095	>10	>10
BA.2.75.1	D574V	0.004	0.479	0.020	>10	0.229	0.141	0.410	>10	0.005	0.002	0.006	0.023	0.057	0.006	4.608	0.208	0.008	0.399	0.003	0.007	0.696	0.200	0.024	0.108	0.248	1.517	0.259	1.660	0.051	>10	0.083	1.851
BA.2.75.2	R346T/F486S/D1199N	0.002	0.266	0.013	>10	0.328	0.297	0.610	>10	0.465	>10	>10	>10	6.404	0.002	1.539	0.123	0.008	0.215	0.159	>10	>10	>10	0.005	0.786	0.327	>10	>10	>10	>10	3.531	>10	>10
BA.2.75.4	L452R	0.007	0.272	0.020	1.120	0.059	0.029	0.097	1.522	0.003	0.001	0.004	0.013	0.031	0.008	5.440	0.266	0.008	0.257	0.004	0.006	3.084	0.053	0.345	1.196	8.823	>10	>10	>10	2.017	1.219	0.056	0.884
BA.2.75.5	K356T	0.002	0.338	0.012	1.410	0.185	0.082	0.164	2.757	0.002	0.001	0.003	0.017	0.091	0.002	2.322	0.197	0.004	2.453	0.003	0.025	>10	>10	0.009	0.042	0.387	>10	0.273	>10	0.070	6.066	0.072	6.696
BA.2.75.6	R346T	0.011	0.336	0.027	0.693	0.076	0.052	0.142	1.180	0.002	0.002	0.005	0.023	0.043	0.004	0.799	0.079	0.009	0.383	0.340	4.524	2.493	5.272	0.008	0.541	1.140	>10	>10	>10	>10	1.175	0.072	0.955
BA.2.75-R403K	R403K	0.009	0.120	0.032	0.577	0.005	0.005	0.030	5.100	0.0003	0.002	0.001	0.009	0.016	0.002	3.903	0.041	0.002	0.504	0.002	0.004	0.540	0.061	0.012	0.014	0.030	0.234	0.059	0.234	0.060	4.421	0.021	0.344
BA.2.75-K444M	K444M	0.006	0.291	0.017	1.943	0.043	0.042	0.089	3.832	0.001	0.001	0.003	0.008	0.019	>10	>10	0.348	0.211	0.183	0.866	2.638	>10	0.149	0.125	>10	0.249	0.513	>10	0.839	>10	6.567	0.040	0.763
BA.2.75-K444T	K444T	0.004	0.207	0.012	0.869	0.039	0.048	0.066	1.036	0.002	0.001	0.003	0.009	0.020	>10	>10	0.458	>10	0.182	0.509	>10	1.852	0.120	0.136	>10	0.124	0.297	>10	0.640	>10	>10	0.048	0.628
BA.2.75-F490S	F490S	0.003	0.261	0.014	1.278	0.098	0.051	0.146	3.598	0.001	0.001	0.003	0.009	0.030	0.003	1.235	0.079	0.004	0.169	0.002	0.004	0.867	0.055	0.018	0.023	5.454	4.844	4.318	5.298	0.034	1.343	0.041	1.163
BA.2.75-D1199N	D1199N	0.008	0.247	0.024	0.827	0.044	0.040	0.089	1.024	0.002	0.002	0.003	0.012	0.020	0.003	0.735	0.095	0.006	0.243	0.003	0.006	0.327	0.107	0.010	0.029	0.077	0.295	0.041	0.504	0.029	3.434	0.035	0.593

<0.01	<0.1	<1	<10	>10

Table S2. Demographics of clinical cohorts in this study. Related to **Figure 4**.

Sample ID	Vaccine type and infected strain	Days post-vaccination or *infection (after last exposure)	Documented COVID-19	Age	Gender	Race/Ethnicity
3 shots WT						
1	mRNA-1273/mRNA-1273/mRNA-1273	29	No	66	Female	White
2	BNT162b2/BNT162b2/BNT162b2	30	No	68	Male	White
3	BNT162b2/BNT162b2/BNT162b2	14	No	64	Female	Asian
4	BNT162b2/BNT162b2/BNT162b2	34	No	55	Male	Asian
5	BNT162b2/BNT162b2/BNT162b2	34	No	45	Male	Asian
6	BNT162b2/BNT162b2/BNT162b2	15	No	48	Female	Asian
7	BNT162b2/BNT162b2/BNT162b2	29	No	71	Male	Asian
8	BNT162b2/BNT162b2/BNT162b2	87	No	66	Female	Asian
9	BNT162b2/BNT162b2/BNT162b2	84	No	26	Male	White
10	mRNA-1273/mRNA-1273/mRNA-1273	23	No	28	Female	White
11	BNT162b2/BNT162b2/mRNA-1273	32	No	39	Male	Asian
3 shots WT + bivalent						
12	BNT162b2/BNT162b2/BNT162b2/Moderna Bivalent	24	No	38	Female	White
13	BNT162b2/BNT162b2/BNT162b2/Moderna Bivalent	27	No	42	Female	White
14	mRNA-1273//mRNA-1273/mRNA-1273/Moderna Bivalent	24	No	36	Male	White
15	BNT162b2/BNT162b2/BNT162b2/Pfizer Bivalent	25	No	37	Female	White
16	BNT162b2/BNT162b2/BNT162b2/Pfizer Bivalent	24	No	36	Male	White
17	BNT162b2/BNT162b2/BNT162b2/Pfizer Bivalent	25	No	49	Female	White
18	BNT162b2/BNT162b2/BNT162b2/Moderna Bivalent	25	No	37	Female	Asian
19	BNT162b2/BNT162b2/BNT162b2/Pfizer Bivalent	26	No	45	Male	White
20	BNT162b2/BNT162b2/mRNA-1273/Moderna Bivalent	26	No	43	Female	White
21	mRNA-1273/mRNA-1273/mRNA-1273/Moderna Bivalent	29	No	32	Female	White
22	BNT162b2/BNT162b2/BNT162b2/Pfizer Bivalent	26	No	43	Female	White
23	BNT162b2/BNT162b2/mRNA-1273/Moderna Bivalent	29	No	38	Female	White
24	BNT162b2/BNT162b2/BNT162b2/Moderna Bivalent	28	No	38	Female	White
25	BNT162b2/BNT162b2/mRNA-1273/Moderna Bivalent	27	No	36	Female	White
26	BNT162b2/BNT162b2/BNT162b2/Moderna Bivalent	30	No	24	Female	White
27	mRNA-1273/mRNA-1273/mRNA-1273/Moderna Bivalent	30	No	32	Female	Asian
28	BNT162b2/BNT162b2/mRNA-1273/Moderna Bivalent	23	No	39	Male	Asian
29	BNT162b2/BNT162b2/BNT162b2/Pfizer Bivalent	30	No	26	Female	Asian
BA.2 breakthrough						
30	BNT162b2/BNT162b2/BA.2	*14	Yes	50	Female	Asian
31	BNT162b2/BNT162b2/BNT162b2/Ad26.COV2.S/BA.2	*22	Yes	69	Male	Asian
32	BNT162b2/BNT162b2/mRNA-1273/BA.2	*16	Yes	32	Male	Asian
33	mRNA-1273/mRNA-1273/mRNA-1273/BA.2	*14	Yes	34	Male	Asian
34	BNT162b2/BNT162b2/mRNA-1273/BA.2	*19	Yes	33	Female	Asian
35	BNT162b2/BNT162b2/mRNA-1273/BA.2	*18	Yes	29	Female	Asian
36	BNT162b2/BNT162b2/monovalent boost/BA.2	*122	Yes	22	Male	White
37	mRNA-1273/mRNA-1273/monovalent boost/BA.2	*164	Yes	30	Female	White
38	BNT162b2/BNT162b2/monovalent boost/BA.2	*94	Yes	30	Female	White
BA.4/5 breakthrough						
39	BNT162b2/BNT162b2/BNT162b2/BA.5	*29	Yes	44	Female	Unknown
40	BNT162b2/BNT162b2/mRNA-1273/BA.5	*29	Yes	36	Female	Asian
41	BNT162b2/BNT162b2/BNT162b2/BNT162b2/BA.5	*31	Yes	54	Female	White
42	BNT162b2/BNT162b2/BNT162b2/BNT162b2/BA.5	*28	Yes	69	Male	White
43	BNT162b2/BNT162b2/BNT162b2/BNT162b2/BA.5	*42	Yes	44	Male	White
44	BNT162b2/BNT162b2/BNT162b2/BNT162b2/BA.5	*28	Yes	41	Female	White
45	BNT162b2/BNT162b2/BNT162b2/BNT162b2/BA.5	*31	Yes	29	Female	White
46	BNT162b2/BNT162b2/BNT162b2/BNT162b2/BA.5	*29	Yes	48	Female	White
47	BNT162b2/BNT162b2/BNT162b2/BNT162b2/BA.5	*29	Yes	49	Female	Asian
48	BNT162b2/BNT162b2/mRNA-1273/BNT162b2/BA.5	*28	Yes	37	Female	White
49	mRNA-1273/mRNA-1273/BNT162b2/BA.5.2.1	*29	Yes	29	Female	White
50	BNT162b2/BNT162b2/BNT162b2/BA.5	*22	Yes	61	Female	White
51	mRNA-1273/mRNA-1273/mRNA-1273/BA.5	*15	Yes	28	Female	White
52	mRNA-1273/mRNA-1273/mRNA-1273/BA.5	*21	Yes	24	Female	White
53	BNT162b2/BNT162b2/BNT162b2/BA.5	*75	Yes	35	Female	White
54	BNT162b2/BNT162b2/mRNA-1273/BA.5	*63	Yes	46	Female	White
55	BNT162b2/BNT162b2/BNT162b2/BA.5	*28	Yes	55	Male	Asian
56	BNT162b2/BNT162b2/BNT162b2/BA.5	*17	Yes	57	Female	White
